# Effects of Multidimensional Self-Esteems on Health Promotion Behaviors in Adolescents

**DOI:** 10.3389/fpubh.2022.847740

**Published:** 2022-04-26

**Authors:** Bin Liu, Lu Tian, Shuo Yang, XueQiang Wang, Jiong Luo

**Affiliations:** College of Physical Education, Southwest University, Chongqing, China

**Keywords:** health promotion school, background factors, multidimensional self-esteem, health promotion behavior, evaluation orientation, association study

## Abstract

**Objective:**

To explore the relationships between multidimensional self-esteems and health behaviors among adolescents by demographic background factors, so as to provide an important reference for the intervention of health promotion behavior and self-esteem education in the future.

**Methods:**

Taking adolescents in Chongqing as the object, this paper investigates the students in 24 primary and secondary schools (half of health promotion schools and half of non-promotion schools) by means of Stratified random sampling, and Using SPSS 21.0 and AMOS 19.0 statistical analysis software to process the collected data.

**Results:**

1) gender and age significantly affected adolescents' self-esteem and health promotion behavior, which showed that boys's perception of self-esteem was lower than that of girls, while girls were more likely to implement health promotion behavior than boys; 2) Children from two parent families or families with higher parental education are more able to implement health promotion behavior and enjoy higher self-esteem, while family economic status has no effect on adolescents' self-esteem and health promotion behavior; 3) Compared with students in ordinary schools, adolescents in health promotion schools (HPS) have higher self-esteem and can implement health promotion behavior more; 4) The higher the self-esteem of adolescents, the better their health promotion behavior; The higher the sense of interpersonal ability, the more able to implement social support behavior; The higher the sense of physical ability and physiological value, the more able to implement sports behavior; The higher the sense of academic ability, the more able to implement nutritional behavior; The higher the external recognition and physiological value, the better the performance of nutritional behavior and stress management; The higher the internal evaluation, the more able to implement health responsibility and exercise behavior.

**Conclusion:**

Socio-economic background can indeed have a direct or indirect impact on adolescent health promotion behavior, and multidimensional self-esteem can explain about 70% of the variation of health promotion behavior, which seems to suggest that improving adolescent self-esteem is the focus of health promotion and health education in the future.

## Introduction

Self-esteem is an individual's emotional experience and evaluation of self-worth formed in the process of socialization. It not only plays an important role in individual development, but also has a significant impact on people's behavior, thinking, emotion, health status, mental health, interpersonal relationship, life satisfaction, motivation, attribution style and achievement performance (1–4). The self-esteem evaluation was first developed by Rosenberg (5), and then further developed by mash (6) into a series of self description questionnaires (SDQ), which pushed the self-esteem construction from unidirectional to multidirectional. At present, SDQ I is applicable to children, SDQ II is applicable to high school adolescents, and SDQ III is applicable to late adolescents and adults (6–9).

The concept of health promotion was first put forward by Pender (10). He believes that health protection and health promotion are more positive methods to achieve healthy behavior, because these two strategies have positive significance of prevention. By integrating the concepts of nursing and behavioral science, Pender (11) developed a “health promotion model” to explain the factors of individual health promotion behavior. The assumption of this model is that individuals have a drive toward health, and their definition of self-health will be more important than generally recognized health status. Individuals will express their unique self through their own cognitive perception patterns and correction factors, And have the ability to adjust themselves to achieve healthy behavior.

The level of adolescents' self-esteem is not only the root of healthy psychology, but also one of the important variables to predict people's health promotion behavior. It is also the focus and key of adolescents' health promotion behavior education. Self-esteem plays an important role whether it is passively avoiding harmful behavior or actively engaging in health promotion behavior. Kheswa study found (12) that subjects' lack of self-consciousness, inappropriate, guilt, shame or inferiority, inability to accept themselves, lack of self-confidence and hatred of themselves are the results of self-esteem distortion. Nihill research shows that (13) adolescents with low self-esteem can't trust others, have poor interpersonal relationships, have a negative view of themselves, and are depressed in academic achievement and achievement motivation. According to Waschull Research (14), even if people with high self-esteem are in a bad state, they can still identify with themselves and do things with positive behavior, while those with low self-esteem often lose self-monitoring and attempt to engage in some prohibited behaviors even if they are in a good and pleasant state. Erol (15) conducted a follow-up study on the overall self-esteem level of more than 7100 American adolescents and found that the overall self-esteem level of European, African and Latin American adolescents showed a steady upward trend from the age of 14 to the early stage of youth. Birkeland et al. (16) found that 87.1% of Norwegian teenagers' overall self-esteem showed a stable upward trend, while 5.5% of the subjects' self-esteem was always at a low level, and 7.4% of the subjects showed a downward trend. Martin et al. found that (17) from middle school to college, teenagers' sense of self-worth generally shows an upward trend. In the third grade of senior high school, students' sense of self-worth decreases significantly, and then gradually increases. By the third grade of college, students' self-esteem in the dimensions of interpersonal relationship and family reaches the highest level. Roelen's research shows that (18), 15 years old and 17−18 years old are the two trough periods of teenagers' self-esteem development. According to Zapata-Lamana et al. research (19), drug abuse, unmarried pregnancy, low academic achievement, campus violence, suicide, truancy, racing and other different ways in adolescent problems are all derived from their deep sense of self frustration, but they are eager to obtain a sense of achievement, so they can only meet their deep self-esteem needs in an abnormal way.

In the research on the relationship between adolescents' self-esteem and their health promotion behavior, Huang showed through meta-analysis of relevant literature (20), the level of individual self-esteem is closely related to emotion (such as anxiety), cognition (such as self-efficacy and academic achievement) and health behavior performance, which shows that high self-esteem plays a positive role in promoting individual psychological development and health behavior. Xiaomiao et al. believe (21) that self-esteem is an individual's experience of self-worth and importance. People with high self-esteem pay high attention to health value, so they have strong health responsibility, while patients with low self-esteem are on the contrary. In the research on College Students' exercise attitude, Luo found (22) that exercise, as a predictive variable, can stimulate individual physical fitness perception and ability perception, promote physical acceptance, enhance self-worth, and then improve the outcome variable self-esteem. Tremblay et al. (23) found that children with high self-esteem also have low average body mass index, and the level of self-esteem can significantly and positively predict the frequency of children's sports activities. Schafer et al. (24) found that after adjusting for variables such as age, education, economic income and body mass index, high self-esteem is still the main predictor of vitamin C and folic acid intake in fruit and vegetable foods for girls (22). Exercise as a predictive variable can stimulate individual physical fitness perception and ability perception, promote physical acceptance, enhance self-worth, and then improve the outcome variable self-esteem. Tremblay et al. (23) found that children with high self-esteem also have low average body mass index, and therefore the level of self-esteem can significantly and positively predict the frequency of children's sports activities. In the research on Adolescent learning satisfaction, Vaquero-Solis et al. found (25) that Youth self-evaluation core (i.e., the value orientation of self-esteem) has the strongest correlation with the dimension of self realization. Rieger et al. (26) believe that when individual self-esteem decreases, self limiting will be triggered out of the protection of self-image. Although it can maintain short-term self-worth, it is not the best strategy to deal with threat situations in the long term. Jambor et al. (27) pointed out that self-esteem plays an intermediary role in behavior regulation in the process of adaptation between individuals and social and cultural environment, and then affects the enthusiasm and initiative of individual interpersonal communication. Fukuya et al. (28, 29) believe that high self-esteem means that individuals have a clear self-concept, which can help individuals establish a health assessment of stress events, avoid adverse emotional disorders such as anxiety or depression, maintain optimistic expectations for the future, reduce tension and conflict in personal life, and gradually adapt to the surrounding environment and self.

Looking at the current research literature on adolescent self-esteem and health promotion behavior by scholars at home and abroad, There are obvious deficiencies as follows: 1) most studies have carried out a series of theoretical construction and related empirical research around self-esteem (such as the exploration of the formation and development of adolescents' early self-esteem; the horizontal and vertical comparison of adolescents' self-esteem characteristics; the research on the relationship between self-esteem of special groups and mental health; the buffering effect of self-esteem on bad emotions in the induced failure situation; the memory bias and emotional response of subjects with different self-esteem types, etc.), and there are few studies on the relationship between adolescents' self-esteem and their health promotion behavior. 2) Rosenberg self-esteem scale (SES) was used (one-way scale) is mostly studied, and the research direction is basically controlled by western scholars, which is dependent to a certain extent and lacks independent innovative thinking; 3) there are many repetitive studies on research methods and content system, and almost no scholars can explore the impact mechanism of multi-dimensional self-esteem on adolescent health promotion behavior. Based on the role of self-esteem in adolescent health promotion in China In terms of the value and role of national physical health education, this study will use the multi-dimensional self-esteem measurement as a tool, through multiple regression and canonical correlation analysis to reveal the relationship between multi-dimensional self-esteem and adolescent health promotion behavior, so as to provide an important reference basis for the intervention of adolescent self-esteem education and health promotion behavior.

## Methods

### Respondents

Firstly, four districts were randomly selected from the nine main urban areas of Chongqing. Then, according to the list of health promotion schools provided by the Municipal Education Commission and sorted according to the distance from the school to the downtown area, two primary schools, two junior middle schools and two senior high schools (Half health promotion schools and half non promotion schools) were randomly selected from each district, and then three classes were randomly selected from the selected schools, A total of 24 the target schools and 72 classes were obtained. Through contact with school leaders and relevant class head teachers, and with the help of relevant subject teachers, complete the questionnaire survey of students in relevant classes.

The survey began on May 10, 2020, and all questionnaires were collected before June 10. A total of 3500 questionnaires were distributed, 240 invalid questionnaires (excluded), 3260 valid questionnaires, with an effective recovery rate of 93.1%. The exclusion criteria are as follows: 1) those whose gender is unknown; 2) those who do not answer key questions. (special note: the exclusion criteria does not mean that all valid samples are fully filled in, but only have no impact on local problems of the study. See Table 1 for the distribution of sample size.

**Table 1:** Distribution of formal survey samples.

	**Sample distribution**	**Gender**			**grade %**		**Total**
		**Male**	**Female**	**Primary school**	**Junior high school**	**High school**	
Jiangbei District	Central urban	201	186	112	135	140	387
	Suburban town	241	174	132	124	159	415
Yuzhong District	Central urban	210	191	129	138	134	401
	Suburban town	207	205	121	154	137	412
Beibei District	Central urban	191	206	148	110	139	397
	Suburban town	206	209	117	154	144	415
South Bank district	Central urban	220	200	122	145	153	420
	Suburban town	230	183	126	115	172	413
Total		1,706	1,554	1,007	1,075	1,178	3,260

### Questionnaire Design

#### Questionnaire Structure

The whole questionnaire consists of basic information of subjects and five scales:

1) The basic data of subjects included seven variables: gender, grade, race, HPS characteristics, family structure, parental education and family economy.
2) On the basis of collecting the relevant theories of self-esteem, self-concept and self-identity at home and abroad, it is sorted out to form the scale framework of “multi-dimensional self-esteem at the connotation of self-esteem and life level”. The connotation of overall self-esteem has four aspects: sense of ability, sense of control, sense of value and evaluation orientation. ability refers to an individual's evaluation and feeling of his abilities in all aspects. Control refers to the degree to which an individual feels or expects to be able to control himself in the face of life affairs. value refers to the degree to which an individual feels valuable, worthy of being loved, and the importance of his own views and feelings. Evaluation orientation refers to the source and method used by individuals to evaluate their self-worth.
3) Eight experts from five physical education departments of colleges and universities in Southwest China were invited to review. The Delphi method was adopted for three rounds at first, followed by two discussion meetings on the items of the scale. After review and discussion, a pre-test scale was formed. After item analysis, the items related to the total score product difference of the subscale that did not reach a significant level were deleted, and finally a formal measurement scale was obtained, including 84 items, That is, the ability subscale has 23 questions, the sense of control subscale 21 questions, the sense of value subscale 22 questions, and the evaluation orientation subscale 18 questions.
4) Health promotion behavior scale. The adolescent health promotion scale developed by Chen (30) was selected. The scale is mainly developed according to Pender's (10) concept of health promotion model, and has been widely used at home and abroad. The scale has 40 questions, including six dimensions: nutritional behavior, interpersonal support, health responsibility, self realization, sports participation and stress management.

All items of the scale are compiled with Likert's five point scale, which are divided into very disagree, disagree, uncertain, agree and very agree, with 1 to 5 points respectively.

#### Questionnaire Validity and Reliability

Table 2 shows:

1) The ability subscale could extract four common factors (KMO = 0.83 and Bartlett's spherical test value reached a significant level (*P* < 0.001), and the cumulative contribution rate of the four common factors could explain 65.82% of the total variation. Cronbach' α coefficient in 4 dimensions is between 0.70–0.82, and the overall scale α Coefficient = 0.84; Confirmatory factor analysis showed that the corresponding values of each fitness index AGFI, CFI, NFI and IFI were 0.93, 0.91, 0.92, and 0.95 respectively, which were greater than the standard of 0.90, RMSEA = 0.036 (< 0.05, good fitness). It can be seen that the reliability and validity of this scale are good.
2) The control subscale could extract four common factors (KMO = 0.78 and Bartlett's spherical test value reached a significant level (*P* < 0.001), and the cumulative explained variation of the four common factors reached 68.00% of the total variation. Cronbach ' α coefficient in 4 dimensions is between 0.71–0.80, and the overall scale α Coefficient = 0.78; Confirmatory factor analysis results revealed that the goodness-of-fit statistics of AGFI, CFI, NFI and IFI were 0.91, 0.94, 0.93 and 0.92 respectively, which were greater than the standard of 0.90, RMSEA = 0.032 (< 0.05, good fitness). It can be seen that the reliability and validity of this scale are good.
3) Four common factors [KMO = 0.84 and Bartlett's spherical test value reached significant (P <0.001)] can be extracted from the sense of value subscale, of which the cumulative explanatory variation of the four common factors reached 61.78% of the total variation. Cronbach 'αcoefficient in dimension 4 of the scale is between 0.74-0.81, and the overall scale α Coefficient = 0.80; Confirmatory factor analysis showed that the corresponding values of each fitness index AGFI, CFI, NFI and IFI were 0.92, 0.90, 0.95 and 0.94 respectively, which were greater than the standard of 0.90, RMSEA = 0.027 (< 0.05, good fitness). It can be seen that the reliability and validity of this scale are good.
4) The evaluation orientation subscale could extract three common factors (KMO = 0.79 and Bartlett's spherical test value was significant (*P* < 0.001), and the cumulative explained variation of the three common factors was 71.60% of the total variation. Cronbach 'α coefficient in dimension 3 of the scale is between 0.71–0.75, and the overall scale α Coefficient = 0.79; Confirmatory factor analysis showed that the corresponding values of each fitness index AGFI, CFI, NFI and IFI were 0.94, 0.93, 0.94 and 0.92 respectively, which were greater than the standard of 0.90, RMSEA = 0.041 (< 0.05, good fitness). It can be seen that the reliability and validity of this scale are good.
5) Six common factors [KMO = 0.77 and Bartlett's spherical test value reached significant (*P* < 0.001)] were extracted from the health promoting life behavior scale (40 questions), and the cumulative explained variation of the six common factors was 67.59% of the total variation. Cronbach 'α coefficient in dimension 6 of the scale is between 0.73–0.81, and the overall scale α Coefficient = 0.80; Confirmatory factor analysis showed that the corresponding values of each fitness index AGFI, CFI, NFI and IFI were 0.95, 0.94, 0.93 and 0.95 respectively, which were greater than the standard of 0.90, RMSEA = 0.022 (< 0.05, good fitness). It can be seen that the reliability and validity of this scale are good.

**Table 2:** Common factor extraction and reliability analysis of adolescent multidimensional self-esteem and health promotion behavior scale.

	**KMO and Bartlet T**	**Common factor naming**	**Items**	**Eigenvalue**	**Explained variation%**	**Progressive interpretation variance%**	**Cronbach α coefficient**
Competency subscale	KMO = 0.83	Sense of family ability	5	7.34	27.45	27.45	0.77
	*P* = 0.000	Sense of physical ability	6	4.75	17.76	45.21	0.72
		Sense of academic ability	6	3.62	13.54	58.75	0.70
		Sense of interpersonal competence	6	1.89	7.07	65.82	0.82
Model verification: the corresponding values of AGFI, CFI, NFI and IFI are 0.93, 0.91, 0.92 and 0.95 respectively; RMSEA=0.036; Overall Cronbach a = 0.84							
Control subscale	KMO = 0.78	Sense of academic control	5	8.37	28.62	28.62	0.75
	*P* = 0.000	Sense of family control	5	6.24	21.34	49.96	0.77
		Sense of control	5	3.47	11.81	61.77	0.71
		a feeling of debility	6	1.88	6.23	68.00	0.80
Model verification: the corresponding values of AGFI, CFI, NFI and IFI are 0.91, 0.94, 0.93 and 0.92 respectively; RMSEA = 0.032; Overall Cronbach a = 0.78							
Sense of value subscale	KMO = 0.84	Interpersonal value	6	7.69	25.27	25.27	0.81
	*P* = 0.000	Family values	6	5.87	19.29	44.56	0.79
		Sense of academic value	5	3.19	10.48	55.04	0.74
		Sense of physiological value	5	2.05	6.74	61.78	0.80
Model verification: the corresponding values of AGFI, CFI, NFI and IFI are 0.5 respectively 92, 0.90, 0.95, 0.94;RMSEA=0.027; Overall Cronbach a = 0.80							
Evaluation orientation subscal	KMO = 0.79	External recognition	6	7.41	35.55	35.55	0.74
	*P* = 0.000	Absolute standard	6	5.40	25.92	61.47	0.71
		Internal evaluation	6	2.11	10.13	71.60	0.75
Model verification: the corresponding values of AGFI, CFI, NFI and IFI are 0.94, 0.93, 0.94 and 0.92 respectively; RMSEA=0.041;Overall Cronbach a = 0.79							
Health promotion behavior scal	KMO = 0.77	Dietary and nutritional behavior	5	9.15	20.76	20.76	0.73
	*P* = 0.000	Health responsibility behavior	8	7.24	16.43	37.19	0.76
		Self fulfilling behavior	8	5.62	12.76	49.95	0.75
		Social support behavior	6	3.71	8.42	58.37	0.81
		Sports participation behavior	4	2.58	5.86	64.23	0.79
		Stress management behavior	9	1.48	3.36	67.59	0.77
Model verification: the corresponding values of AGFI, CFI, NFI and IFI are 0.95, 0.94, 0.93 and 0.95 respectively; RMSEA=0.022; Overall Cronbach a = 0.80							

### Mathematical Statistics

Classify and code the collected data according to the scale items, and establish a database. Use spss21.0 and amos19.0 statistical analysis software was used to combine exploratory (EFA) and confirmatory (CFA) factor analysis, and cluster analysis, multiple linear regression and canonical correlation were added to explore the correlation between adolescents' personal background factors, multidirectional self-esteem and health behavior. The significance level of all indicators was set as a = 0.05.

## Results

### Characteristics of Health Promotion Behavior by Demographic Background Factors

The personal background factors of adolescents in this study mainly include gender, grade, race, HPS, family structure, parental education, family economy and so on. Taking personal background variables as independent variables and adolescent health promotion behavior as dependent variables, stepwise elimination method was used for multiple linear regression analysis. First, virtualize five variables such as gender, grade, race, HPS and family structure, Gender (reference category: girl), grade (reference category: primary school), race (reference category: other ethnic groups), HPS (reference category: ordinary schools), family structure (reference category: other family models), parental education and family economy are continuous variables and need not be virtualized. The information of the seven regression equations in Table 3 is as follows:

**Table 3:** Statistical table of regression equation for the influence of adolescents' personal background factors on their health promotion behavior.

**Regression equation (Y)**	**Gender**	**Grade**	**Race**	**HPS characteristics**	**Family structure**	**Parental education**	**Family economy**	**Coefficient of determination**
	**f1**	**f2**	**f3**	**f4**	**f5**	**f6**	**f7**	**(R^**2**^)**
(A) self-realization		0.50^**^		0.28^**^	0.61^**^			0.12
(B) Health responsibility	−0.49^**^			0.51^**^	0.59^**^	0.16^*^		0.22
(C) Stress management	0.39^**^	0.36^**^			0.48^**^	−0.31^**^		0.09
(D) Social support	0.44^**^	−0.33^**^		0.51^**^	0.55^**^			0.13
(E) Nutritional behavior	−0.35^**^			0.52^**^	0.46^**^			0.18
(F) Sports participation	0.33^**^	−0.39^**^		0.47^**^	0.41^**^			0.24
(G) Overall promotion	0.26^**^			0.59^**^	0.68^**^	0.29^**^		0.17

*, ** *represent the statistical significance levels of 0.05, 0.01 and 0.001 respectively*.

Regression equation (A) means the influence model of teenagers' background factors on self realization behavior. Three background factors were introduced (four were eliminated). They are grade, school characteristics and family structure respectively. The corresponding standardized regression coefficients are 0.50^**^, 0.28^**^, and 0.61^**^, and the regression equation has reached a significant level (*P* < 0.01), R^2^ = 0.12, indicating that the introduction of three background factors can explain 12.00% of the variation of teenagers' self realization behavior; the three standardized coefficients are positive, indicating that their influence is positive, which means that middle school students than primary school students, students in HPS than ordinary school students and students in two parent families than students in other combination mode families, are easier to promote self realization behavior. From the perspective of the three standardized coefficients, the largest contribution to self realization behavior is family structure, followed by grade, and HPS rank third.

Regression equation (B) represents the influence model of adolescent background factors on health responsibility behavior. Four background factors are introduced, namely gender, HPS, family structure and parental education. The corresponding standardized regression coefficients are −0.49^**^, 0.51^**^, 0.59^**^, and 0.16^*^, and the regression equation has reached a significant level (*P* < 0.01), R^2^ = 0.22, indicating that the introduced four background factors can explain 22.00% of the variation of adolescent health responsibility behavior; among the four standardized coefficients, the coefficient corresponding to gender factor is negative, indicating that compared with girls, boys' health responsibility behavior is worse, and the other three coefficients are positive, which means that the higher the HPS, two parent family and two parent education level is Adolescents have better health responsibility behavior; From the absolute values of the four standardized coefficients, the order of contribution to health responsibility behavior from large to small is family structure, HPS, gender and parental education level.

Regression equation (C) represents the influence model of adolescent background factors on stress management behavior. Four background factors are introduced, namely gender, grade, family structure and parental education. The corresponding standardized regression coefficients are 0.39^**^, 0.36^**^, 0.48^**^, and −0.31^**^, and the regression equation has reached a significant level (*P* < 0.01), R^2^ = 0.09, indicating that the introduced four background factors can explain 9.00% of the variation of adolescents' stress management behavior; among the four standardized coefficients, the coefficient corresponding to parental education factors is negative, which means that the higher the total number of years of education, the higher the adolescents' stress management ability The worse the (stress release), this finding is surprising! The other three coefficients are positive, which means that men, middle school students and adolescents from two parent families have better stress management behavior; from the absolute values of the four standardized coefficients, the contributions to stress management behavior are family structure, gender, grade and two parent education.

Regression equation (D) represents the influence model of adolescent background factors on social support behavior. Four background factors are introduced, namely gender, grade, HPS and family structure. The corresponding standardized regression coefficients are 0.44^**^, −0.33^**^, 0.51^**^, and 0.55^**^, and the regression equation has reached a significant level (*P* < 0.01), R^2^ = 0.13, indicating that the introduced four background factors can explain 13.00% of the variation of teenagers' social support behavior; among the four standardized coefficients, the coefficient corresponding to grade factor is negative, which means that middle school students are inferior to primary school students in obtaining social support, and the other three coefficients are positive, which shows that men, students from HPS and children from two parent families are easier to obtain social support More social support; In terms of the absolute values of the four standardized coefficients, the contribution to social support behavior from large to small is family structure, HPS, gender and grade.

Regression equation (E) represents the influence model of adolescent background factors on nutritional behavior. Three background factors were introduced, namely gender, HPS and family structure. The corresponding standardized regression coefficients were −0.35^**^, 0.52^**^, and 0.46^**^, and the regression equation reached a significant level (*P* < 0.01), R^2^ = 0.18, indicating that the three background factors introduced can explain 18.00% of the variation of teenagers' nutritional behavior; among the three standardized coefficients, the coefficient corresponding to gender factor is negative, which means that boys' nutritional behavior is worse than girls, and the other two coefficients are positive, which shows that students in HPS and children from two parent families have better nutritional behavior; the three standardized coefficients are absolute From the perspective of value, the order of contribution to adolescent nutritional behavior from large to small is HPS, family structure and gender.

The regression equation (F) represents the influence model of teenagers' background factors on sports participation behavior. Four background factors are introduced, namely gender, grade, HPS and family structure. The corresponding standardized regression coefficients are 0.33^**^, −0.39^**^, 0.47^**^, and 0.41^**^, and the regression equation has reached a significant level (*P* < 0.01), R^2^ = 0.24, indicating that the introduced four background factors can explain 24.00% of the variation of teenagers' sports participation behavior; among the four standardized coefficients, the coefficient corresponding to grade factor is negative, which means that middle school students' sports participation behavior is worse than primary school students, and the other three departments are positive, which shows that boys, students from HPS and children from two parent families have better sports participation behavior Active participation behavior; In terms of the absolute values of the four standardized coefficients, the order of contribution to teenagers' sports participation behavior from large to small is HPS, family structure, grade and gender.

Regression equation (G) represents the overall impact model of adolescent background factors on health promotion behavior. Four background factors are introduced, namely gender, HPS, family structure and parental education. The corresponding standardized regression coefficients are 0.26^**^, 0.59^**^, 0.68^**^, and 0.29^**^, and the regression equation has reached a significant level (*P* < 0.01), R^2^ = 0.17, indicating that the introduced four background factors can explain 17.00% of the variation of adolescents' overall health promotion behavior; the four standardized coefficients are positive, which shows that boys, students in HPS, children from two parent families and children from families with high parental education level have better health promotion behavior; the absolute values of the four standardized coefficients are significant for adolescents as a whole The order of health promotion behavior contribution from large to small is family structure, HPS, parental education and gender.

### Influence of Background Factors on Adolescents' Multidimensional Self-Esteem

Adolescent self-esteem consists of four subscales—sense of ability, sense of control, sense of value and evaluation orientation. Firstly, sum the dimensions of the four subscales to calculate the total score, then calculate the total score of the self-esteem scale, and finally explore the influence of teenagers' personal background factors on the five aspects of self-esteem. A total of five regression equations are obtained, as shown in Table 4, from which the following information can be obtained:

**Table 4:** Statistical table of regression equation of the influence of adolescents' personal background factors on their multidirectional self-esteem.

**Regression equation (Y)**	**Gender**	**Grade**	**Race**	**HPS characteristics**	**Family structure**	**Parental education**	**Family economy**	**Coefficient of determination**
	**f1**	**f2**	**f3**	**f4**	**f5**	**f6**	**f7**	**(R^**2**^)**
(H) Sense of ability		0.26^**^		0.31^**^	0.39^**^	0.24^**^	0.19^**^	0.17
(I) Sense of control	0.18^*^	0.34^**^			0.41^**^			0.14
(J) Sense of value				0.23^**^	0.37^**^	0.21^**^		0.25
(K) Evaluation orientation	−0.24^**^	0.33^**^		0.35^**^	0.44^**^	0.20^**^		0.16
(M) Overall self-esteem	−0.17^*^	0.28^**^		0.41^**^	0.37^**^	0.22^**^		0.29

*, ** *represent the statistical significance levels of 0.05, 0.01 and 0.001 respectively*.

Regression equation (H) represents the influence model of teenagers' background factors on ability perception. Five background factors are introduced, which are grade, HPS, family structure, parent education and family economy. The corresponding standardized regression coefficients are 0.26^**^, 0.31^**^, 0.39^**^, 0.24^**^, and 0.19^*^, and the regression equation has reached a significant level (*P* < 0.01), R^2^ = 0.17, indicating that the five background factors introduced can explain 17.00% of the variation of teenagers' sense of ability; the five standardization coefficients are positive, indicating that middle school students, students from HPS, children from two parent families, those with high level of parental education and those with good family economic conditions have a strong sense of self-ability; From the absolute value of the five standardized coefficients, the contribution to teenagers' sense of ability from large to small is family structure, HPS, grade, parent education and family economy.

Regression equation (I) represents the influence model of adolescent background factors on control perception. Three background factors are introduced, namely gender, grade and family structure. The corresponding standardized regression coefficients are 0.18^*^, 0.34^**^, and 0.41^**^, and the regression equation reaches a significant level (*P* < 0.01), R^2^ = 0.14, indicating that the three background factors introduced can explain 14.00% of the variation of teenagers' sense of control; the three standardized coefficients are positive, indicating that boys, middle school students and students from two parent families have a strong sense of self-control; in terms of the absolute values of the three standardized coefficients, the contributions to teenagers' sense of control are family structure, grade and gender in descending order.

The regression equation (J) represents the influence model of teenagers' background factors on value perception. Three background factors are introduced, namely HPS, family structure and parental education. The corresponding standardized regression coefficients are 0.23^**^, 0.37^**^, and 0.21^**^, and the regression equation reaches a significant level (*P* < 0.01), R^2^ = 0.25, indicating that the three background factors introduced can explain 25.00% of the variation of teenagers' sense of value; the three standardization coefficients are positive, indicating that students in HPS, parents and family children with high parental education level have a strong sense of self-worth; From the absolute value of the three standardized coefficients, the contribution to teenagers' sense of self-worth from large to small is family structure, HPS and parental education.

The regression equation (k) represents the influence model of adolescents' background factors on evaluation orientation. Five background factors are introduced, namely gender, grade, HPS, family structure and parental education. The corresponding standardized regression coefficients are −0.24^**^, 0.33^**^, 0.35^**^, 0.44^**^, and 0.20^**^, and the regression equation has reached a significant level (*P* < 0.01), R^2^ = 0.16, indicating that the five background factors introduced can explain 16.00% of the variation of teenagers' evaluation orientation; the standardization coefficient corresponding to gender factors is negative, indicating that girls have a strong evaluation orientation than boys, and the other four are positive, which shows that middle school students, students in HPS, parents and family children with high parental education level have a stronger evaluation orientation; The absolute values of the five standardized coefficients show that the order of contribution to teenagers' evaluation orientation from large to small is family structure, HPS, grade, gender and parental education.

The regression equation (m) represents the influence model of adolescent background factors on overall self-esteem. Five background factors are introduced, namely gender, grade, HPS, family structure and parental education. The corresponding standardized regression coefficients are −0.17^*^, 0.28^**^, 0.41^**^, 0.37^**^, and 0.22^**^, and the regression equation has reached a significant level (*P* < 0.01), R^2^ = 0.29, indicating that the five background factors introduced can explain 29.00% of the variation of adolescents' overall self-esteem; the standardization coefficient corresponding to gender factors is negative, indicating that girls have stronger overall self-esteem perception than boys, and the other four are positive, which shows that middle school students, students in HPS, parents and family children with high parental education level have stronger overall self-esteem Physical self-esteem; The absolute values of the five standardized coefficients show that the order of contribution to adolescents' overall self-esteem from large to small is HPS, family structure, grade, parental education and gender.

### Typical Correlation Analysis Between Multidimensional Self-Esteem and Health Promotion Behavior

Canonical correlation analysis is a statistical method used to test the correlation degree between one group of control variables and another group of criterion variables. The purpose is to find the maximum correlation between the linear combination of control variables and the linear combination of criterion variables. Therefore, canonical correlation analysis tests the canonical correlation combination of multiple criterion variables and multiple control variables, Canonical correlation analysis can produce canonical correlation combinations with significant and insignificant correlation. Generally, it can provide the following basic information: one is the typical correlation coefficient. It can reflect the correlation degree between the linear combination of control variables and the linear combination of standard variables. The typical correlation coefficient must reach the significant level to represent the significant correlation between the two groups of linear combinations. The second is the judgment coefficient (i.e., the square value of the typical correlation coefficient R). It means that the typical factors of the standard variable can be explained by the typical factors of the control variable (not <10%). The third is the structure coefficient (typical load). It means to control the correlation between the variable and the standard variable to their respective typical linear combinations. The absolute value of the coefficient must be more than 0.30 to explain their respective typical linear combinations.

**Figure 1:**
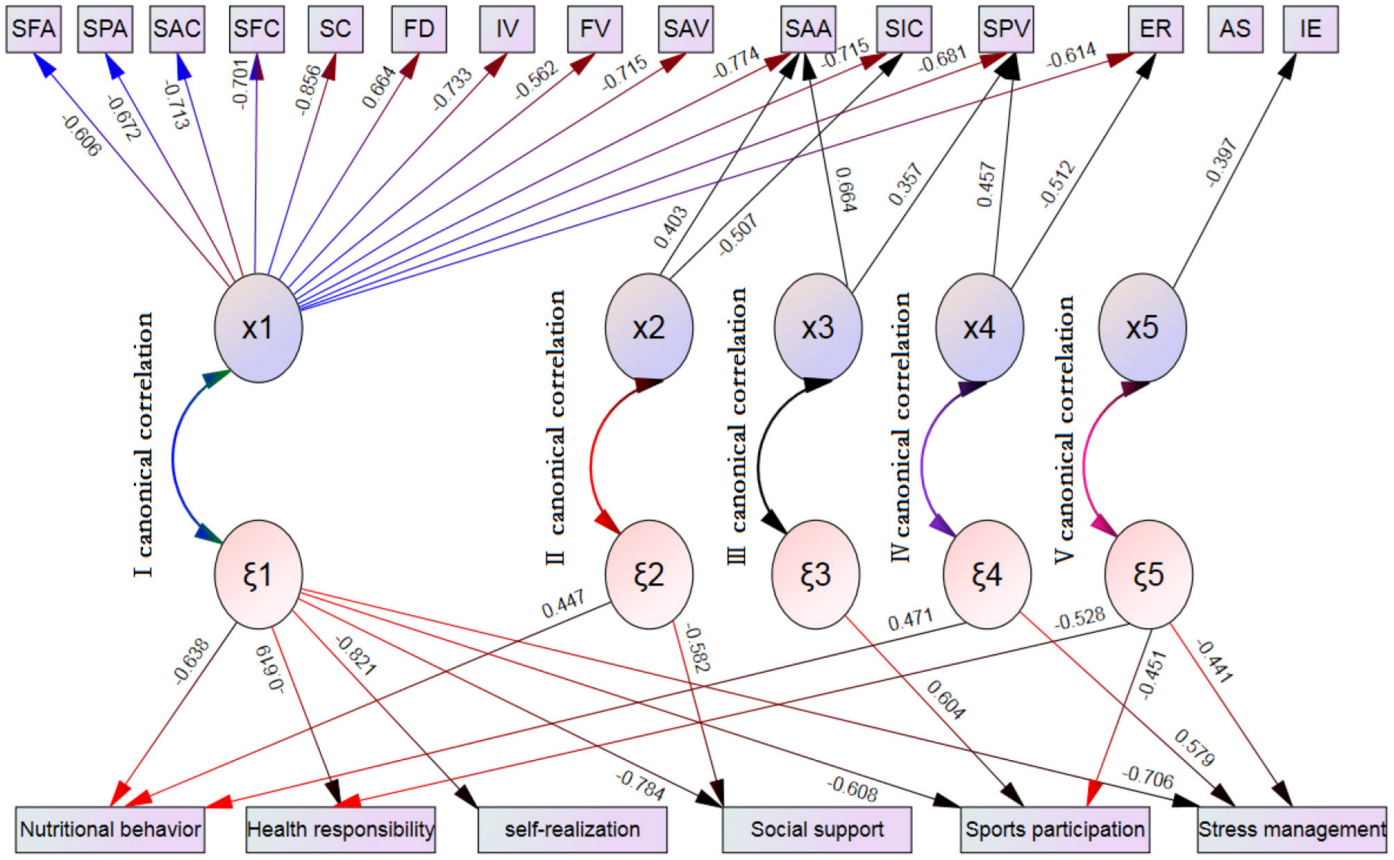
Typical correlation between multidirectional self-esteem and health promotion behavior of teenagers. Only variables with typical factor load higher than 0.30 are retained in the structure diagram, and positive and negative signs relevant directionality. SFA, Sense of family ability; SPA, Sense of physical ability; SSA, Sense of academic ability; SIC, Sense of family interpersonal competence; SAC, Sense of academic control; SFC, Sense of family control; SC, Sense of control; FD, a feeling of debility; IV, Interpersonal value; FV, Family values; SAV; Sense of academic value; SPV, Sense of physiological value; IE, Internal evaluation; ER, External recognition; AS, Absolute standard.

According to the information in Table 5 and Figure 1:

**Table 5:** Typical correlation analysis of multidimensional self-esteem and health promotion behavior.

**X variable**	**Typical factors**					**Y variable**	**Typical factors**				
	**x_**1**_**	**x_**2**_**	**x_**3**_**	**x_**4**_**	**x_**5**_**		**ξ_1_**	**ξ_2_**	**ξ_3_**	**ξ_4_**	**ξ_5_**
Sense of family ability	–**0.606**	−0.204	−0.024	0.175	0.077	Nutritional behavior	–**0.638**	**0.447**	0.281	**0.471**	0.206
Sense of physical ability	–**0.672**	0.218	**0.664**	0.116	−0.123	Health responsibility	–**0.619**	−0.168	−0.077	−0.116	–**0.528**
Sense of academic ability	–**0.774**	**0.403**	−0.137	−0.204	0.176	self-realization	–**0.821**	0.251	−0.217	−0.155	0.072
Sense of interpersonal competence	–**0.715**	–**0.507**	0.127	0.169	0.059	Social support	–**0.784**	–**0.582**	0.116	0.077	0.065
Sense of academic control	–**0.713**	0.227	−0.031	−0.018	−0.054	Sports participation	–**0.608**	0.249	**0.604**	−0.087	–**0.451**
Sense of family control	–**0.701**	−0.057	0.012	−0.020	0.058	Stress management	–**0.706**	0.228	−0.177	**0.579**	–**0.441**
Sense of control	–**0.856**	0.081	−0.156	0.213	−0.289						
a feeling of debility	**0.664**	0.019	0.217	−0.189	0.108						
Interpersonal value	–**0.733**	0.043	−0.072	−0.074	−0.175						
Family values	–**0.562**	0.157	−0.071	0.083	0.232						
Sense of academic value	–**0.715**	0.291	−0.169	0.063	0.145						
Sense of physiological value	–**0.581**	0.206	**0.357**	**0.457**	0.247						
Internal evaluation	–**0.614**	0.151	−0.115	0.054	–**0.397**						
External recognition	0.051	0.271	−0.082	–**0.512**	−0.055						
Absolute standard	0.209	0.265	−0.147	0.237	0.258						
Extraction variance%	43.56	8.58	7.67	7.15	5.27		48.50	8.88	7.79	7.84	9.87
Overlap variance%	21.8	7.86	5.77	3.56	3.01		31.61	5.15	3.68	2.81	2.03
R^2^							0.77	0.38	0.17	0.12	0.10
R							0.87^**^	0.62^**^	0.41^**^	0.35^**^	0.31^**^

*, ** *represent the statistical significance levels of 0.05, 0.01 and 0.001 respectively*.

There are five canonical correlations. The first group of canonical correlations mainly shows the correlation between overall self-esteem and overall health promotion behavior; The second group of canonical correlation mainly explained the correlation between the sense of interpersonal ability and academic ability, nutritional behavior and social support; The third group mainly explained the correlation between physical ability, physiological value and sports participation behavior; The fourth group of canonical correlation mainly explained the correlation between physiological value, external recognition, nutritional behavior and stress management; The fifth group mainly explains the correlation between internal evaluation and health responsibility, sports participation and stress management behavior.

#### Correlation Structure Between Overall Self-Esteem and Overall Health Promotion Behavior

In the first group of canonical correlations, the canonical correlation coefficient *R* = 0.87^**^, and the determination coefficient R^2^ = 0.77, indicating that the first canonical factor (x_1_) of the X variable group can explain the first canonical factor of the Y variable group (ξ_1_) 77% of the total variation; x_1_ is the first typical factor extracted from X variable group, accounting for 43.56% of the total variation of X variable group, and the first typical factor of X variable group and Y variable group(ξ_1_) the overlapping part is 21.8%, which represents the first typical factor of the Y variable group ξ_1_ can explain 21.8% of the total variation of X variable group. and ξ _1_ is the first typical factor extracted from the Y variable group, accounting for 48.5% of the total variation of the Y variable group. The overlapping variation between the Y variable group and the first typical factor (x_1_) extracted from the X variable group is 31.6%, indicating that the first typical factor x_1_ of the X variable group can explain 31.6% of the total variation of the Y variable group.

In the first group of canonical correlation structure, in the X variable group, family ability, physical ability, academic ability, academic ability, family ability, control, powerlessness, interpersonal ability, family value, academic ability, physiological value, internal evaluation, external evaluation and absolute standard are highly correlated with the first canonical factor x_1_, The typical factor loads are −0.606, −0.672, −0.774, −0.715, −0.713, −0.701, 0.856, 0.664, −0.733, −0.562, −0.715, −0.581 and −0.614 respectively. Therefore, the first typical correlation between the X variable group and the Y variable group means that the X variable group mainly depends on the sense of family ability, physical ability, academic ability, academic ability, family ability, control, powerlessness, interpersonal ability, family value, academic ability, physical value, internal evaluation, External evaluation and absolute standard affect the first typical factor ξ_1_ of Y variable group through the first typical factor x1. The variables highly correlated with ξ_1_ were nutritional behavior, health responsibility, self realization, social support, sports participation and stress management, and the corresponding factor loads were −0.638, −0.619, −0.821, −0.784, −0.608 and −0.706, respectively. From the positive and negative signs of factor load, except that the relationship of powerlessness is reverse, the other relationships are in the same direction.

#### The Correlation Structure Between Interpersonal Competence, Academic Competence, Nutritional Behavior and Social Support Behavior

In the second group of canonical correlations, the canonical correlation coefficient *R* = 0.56^**^, and the determination coefficient R^2^ = 0.38, indicating that the second canonical factor (x_2_) of the X variable group can explain the second canonical factor of the Y variable group(ξ_2_) 38% of the total variation; x_2_ is the second typical factor extracted from X variable group, accounting for 8.58% of the total variation of X variable group, and Overlapping variation of the second typical factor of X variable group and Y variable group(ξ_2_) is 7.86%, which indicates that the second typical factor in the Y variable group can explain 7.86% of the total variation in the X variable group. and ξ_2_ is the second typical factor extracted from the Y variable group, accounting for 8.88% of the total variation of the Y variable group. The overlapping variation between the Y variable group and the second typical factor (x_2_) extracted from the X variable group is 5.15%, indicating that the second typical factor x_2_ in group X can explain 5.15% of the total variation of the Y variable group.

From the typical correlation structure of the second group, in the X variable group, the sense of academic ability and interpersonal ability are highly correlated with the second typical factor x_2_, and the load of typical factors is 0.403 and −0.507 respectively. Therefore, the second canonical correlation can be explained that the sense of academic ability and interpersonal ability in the X variable group affect the second canonical factor ξ_2_ in the Y variable group through its canonical factor x_2_. And the health promotion behaviors highly correlated with ξ_2_ were nutritional behavior and social support behavior, and the corresponding factor loads were 0.447 and −0.582, respectively. From the positive and negative symbols of factor load, the relationship between academic ability and nutritional behavior is the same direction, the relationship between interpersonal ability and social support is the same direction, while interpersonal ability and nutritional behavior are reverse, and academic ability and social support behavior are reverse.

#### The Related Structures of Physical Ability, Physiological Value and Sports Participation Behavior

In the third group of canonical correlation, the canonical correlation coefficient *R* = 0.41^**^, and the determination coefficient R^2^ = 0.17, indicating that the third canonical factor (x3) of the X variable group can explain 17% of the total variation of the third canonical factor of the Y variable group; x3 is the third canonical factor extracted from the X variable group, accounting for 7.67% of the total variation of the X variable group, and Overlapping variation of the third typical factor of X variable group and Y variable group(ξ3) is 5.77%, which indicates that the third typical factor in the Y variable group can explain 5.77% of the total variation in the X variable group. and ξ3 is the third typical factor extracted from the Y variable group, accounting for 7.79% of the total variation of the Y variable group. The overlapping variation between the Y variable group and the third typical factor (x3) extracted from the X variable group is 3.68%, indicating that the third typical factor x3 in group X can explain 3.68% of the total variation of the Y variable group.

From the typical correlation structure of the third group, the sense of physical ability and physiological value are highly correlated with the third typical factor x_3_ extracted from the X variable group, and the load of typical factors are 0.664 and 0.357 respectively. Therefore, the third canonical correlation can be explained as that the sense of physical ability and physiological value in the X variable group can affect the third canonical factor ξ_3_ in the Y variable group with the help of the third canonical factor x_3_, while the health promotion behavior highly related to ξ_3_ is sports participation behavior, and its corresponding factor load is 0.604. From the positive and negative symbols of factor load, the sense of physical ability The relationship between physiological sense of value and sports participation behavior is the same direction.

#### External Recognition, Physiological Value, Nutritional Behavior and Stress Management

In the fourth group of canonical correlation, the canonical correlation coefficient R = 0.35^**^, and the determination coefficient R^2^ = 0.12, indicating that the fourth canonical factor x_4_ of the X variable group can explain the fourth canonical factor of the Y variable group(ξ_4_) 12% of the total variation, while x_4_ is the fourth typical factor extracted from X variable group, which accounts for 7.15% of the total variation of X variable group. In addition, the overlapping variation of the fourth typical factor ξ_4_ of Y variable group and X variable group is 3.56%, which means that the fourth typical factor ξ_4_ of Y variable group can explain 3.56% of the total variation of X variable group. However, ξ_4_ is the fourth typical factor extracted from the Y variable group, accounting for 7.84% of the total variation of the Y variable group, while the overlapping variation between the fourth typical factor x_4_ of X variable group and the Y variable group is 2.81%, indicating that the fourth typical factor x_4_ of the X variable group can explain 2.81% of the total variation of the Y variable group.

From the perspective of the typical correlation structure of the fourth group, the sense of physiological value and external recognition are highly correlated with the typical factor x_4_ in the X variable group, and their typical factor loads are 0.457 and −0.512 respectively. Therefore, it can be considered that the sense of physiological value and external recognition in the X variable group affect the fourth typical factor ξ_4_ in the Y variable group through the fourth typical factor x_4_, The health promotion behaviors highly correlated with ξ_4_ were nutritional behavior and stress management behavior, and the corresponding typical factor loads were 0.471 and 0.579 respectively. In addition, from the positive and negative symbols of factor load, the relationship between physiological sense of value and nutritional behavior and stress management is the same direction, while the relationship between external recognition and nutritional behavior and stress management is the opposite.

#### Internal Evaluation and Related Structures of Responsible Behavior, Sports Behavior and Stress Management

In the fifth group of canonical correlation, the canonical correlation coefficient R = 0.31 and the determination coefficient R^2^ = 0.10, indicating that the fifth canonical factor x_5_ in the X variable group can explain 10% of the total variation of the fifth canonical factor ξ_5_ in the Y variable group. x_5_ is the fifth typical factor extracted from the X variable group, accounting for 5.27% of the total variation of the X variable group, of which the overlapping variation of the fifth typical factor ξ_5_ of the Y variable group and the X variable group is 3.01%, indicating that the fifth typical factor ξ_5_ of the Y variable group can explain 3.01% of the total variation of the X variable group. ξ_5_ is the fifth typical factor extracted from the Y variable group, accounting for 9.87% of the total variation of the Y variable group, while the overlapping variation of the fifth typical factor x_5_ of the X variable group and the Y variable group is 2.03%, indicating that the fifth typical factor x_5_ of the X variable group can explain 2.03% of the total variation of the Y variable group.

From the typical correlation structure of the fifth group, in the X variable group, the internal evaluation is highly correlated with the fifth typical factor x_5_, and its typical factor load is −0.397. Therefore, it can be considered that the fifth typical factor x_5_ in the X variable group mainly depends on the internal evaluation to affect the fifth typical factor ξ_5_ in the Y variable group, The fifth typical related factor ξ_5_ from the Y variable group is highly correlated with health responsibility behavior, exercise participation behavior and stress management behavior in health promotion behavior, and the corresponding typical factor loads are −0.528, −0.451 and −0.441 respectively. From the positive and negative signs of factor load, the relationship between internal evaluation and health responsibility behavior, sports participation behavior and stress management behavior is the same direction.

## Discussion

### From the Impact of Gender and Grade on Adolescents' Self-Esteem and Health Promotion Behavior

This study found that: adolescent gender factors have a great impact on their health promotion behavior. Except that self realization behavior is not affected by gender, the other five aspects and overall health promotion are affected by gender. Among them, girls are easier to complete the promotion of health responsibility and nutritional behavior than men; In the three behaviors of stress management, social support and sports participation, men are easier to complete the promotion work; From the perspective of overall health promotion behavior, boys are more likely to complete health promotion than girls. These findings are similar to the results of many previous studies (31–33). This study also found that gender also affects adolescents' self-esteem, in which men have higher control perception than girls, while girls have higher evaluation orientation than men. From the overall self-esteem situation, men's self-esteem perception is lower than girls's; Adolescents' self-esteem and health promotion behavior are also widely affected by grades. In health promotion behavior, middle school students have more advantages than primary school students in four aspects: health responsibility, stress management, social support and sports participation behavior; The impact on adolescent self-esteem is that middle school students have higher ability perception, higher control perception and higher evaluation orientation than primary school students. These findings also support the basic views of many previous scholars (34, 35).

### From the Perspective of the Impact of Family Structure on Adolescents' Self-Esteem and Health Promotion Behavior

This study found that family relations significantly affect young people's self-esteem and health promotion behavior, which shows that children from two parent families are better than single parent and other forms of combined families in the six dimensions of health promotion behavior, and also show the same positive effect in self-esteem, that is, children from two parent families have a sense of ability, control, value evaluation orientation and overall self-esteem were significantly better than those of single parents and other combined families. Some studies believe that (36, 37) children growing up in two parent families have a safer relationship with their parents in the growth process, so they have higher self-esteem and can better cultivate independent ideology, career choice and interpersonal relationships. Those children living in single parent or other combined families often show a stagnant or chaotic identification tendency, Their decision-making lacks self-confidence and self-identity. Other scholars believe that (38, 39), growing up in single parent or other combined families, teenagers will face higher health risks, such as frequent skipping breakfast, higher running away rate, higher detection rate of ill health, high saturated fat and high sodium in food; Most adolescents living with grandparents and uncles or aunts have poor health-related behavior and low self-esteem. The results obtained in this study are similar to those of many scholars mentioned above.

### From the Influence of Parents' Education Level and Family Economic Status on Adolescents' Self-Esteem and Health Promotion Behavior

This study found that parental education plays an important role in adolescent health promotion and self-esteem. Children from families with higher parental education have better health responsibility behavior and adverse stress management behavior; The higher the parents' education level, the higher the children's sense of ability, value, evaluation orientation and overall self-esteem. Previous studies believe that (40–42), there is a significant positive correlation between parents' education level and adolescents' health-related behavior, which supports the relevant conclusions of this study in some aspects. In fact, it is completely understandable that well-educated parents can better convey more health information to their children, which is beneficial to the cultivation of children's health responsibility behavior. However, it is also difficult for well-educated parents to face pressure management. This conclusion is puzzling? The reasons need to be further discussed. In addition, this study also found that family economic status has no effect on adolescent health promotion behavior and overall self-esteem, which is similar to the research results of Wilson et al. (43).

### From the Impact of Health Promotion Schools (HPS) on Adolescents' Self-Esteem and Health Promotion Behavior

This study found that HPS had a significant effect on promoting the cultivation of adolescent health behavior and improving self-esteem. All HPS students were significantly better than ordinary schools in the five dimensions of health promotion (except stress management behavior); The same rules are shown in the sense of ability, sense of value, evaluation orientation and overall self-esteem. As the prominent feature of HPS is to take “enhancing students' physique and promoting students' physical and mental health” as an important starting point of education, it has strict requirements and specific assessment indicators in fund guarantee, sports teacher training, sports venue construction, sports talent training, etc. Students who grow up in HPS have more interpersonal interactions (such as participation in extracurricular activities, sense of belonging, trust, etc.) and play more roles than students in ordinary schools. Therefore, compared with similar children, children with HPS score higher in self realization, stress management, health responsibility, social support, nutritional behavior, sports participation, etc, and they feel that have a greater sense of ability, value and higher evaluation orientation.

### From the Relationship Between Adolescents' Self-Esteem and Their Health Promotion Behavior

The first group of canonical correlations showed that there was a significant correlation between adolescents' overall self-esteem and their overall health promotion behavior in Southwest China, and the explanatory amount was as high as 77%. In other words, the higher the self-esteem, the higher the implementation of health promotion behavior. From the perspective of self-esteem, if adolescents have a high sense of interpersonal ability, physical ability, family ability, academic ability, family control, control, academic control, internal evaluation, interpersonal value, physiological value, family value and academic value, the higher the implementation of their health promotion behavior, and the higher the sense of powerlessness, The more difficult it is to implement health promotion behavior.

The second group of canonical correlations shows that adolescents' social support behavior is mainly affected by their sense of interpersonal competence. The higher their sense of interpersonal competence, the more they can implement social support behavior. This finding is basically consistent with Mohammadzadeh 's research (44), that is, self-esteem can strengthen their adaptability to problems and improve their adaptability to the external environment, Therefore, it is one of the key factors of personal success. Relevant studies also pointed out that (3, 45), people with high self-esteem have self-confidence and like themselves, feel that they are valuable and capable, are willing to take risks and are not afraid of failure, will make persistent efforts when they encounter difficulties, will not worry that others do not like themselves, can treat and accept others kindly, and are willing to help and praise others, Be able to establish good interpersonal relationships with others. However, from the perspective of correlation, there is a significant negative correlation between social support behavior and academic ability. This may be because teenagers bear great academic pressure (entering a higher school). Those students who concentrate on their schoolwork and obtain a sense of achievement in their studies lack attention to interpersonal interaction because they have no time to establish interpersonal relationships or devote themselves to learning. Otherwise, it is difficult to explain this phenomenon, What are the reasons for the need for in-depth clarification? Another typical related finding of this group is that adolescents' nutritional behavior is mainly affected by their sense of academic ability. The higher their sense of academic ability, the more they can implement nutritional behavior. Because the meaning of the sense of academic ability in this study is mainly aimed at the evaluation and feeling of adolescent individuals on their academic ability, people with positive sense of ability have confidence in their ability and performance (46). The cultivation of nutrition related knowledge and health behavior is one of the key contents of adolescent physical education and health curriculum in China. Therefore, adolescent nutrition behavior should be affected by their sense of healthy academic ability. The higher the sense of healthy academic ability, the more able to implement nutrition behavior. Nutritional behavior is also affected by the sense of interpersonal competence, which is negatively correlated, that is, students with good sense of interpersonal competence do not perform well in the implementation of nutritional behavior? The reason may be that teenagers are in a period of emotional transformation, from relying on their elders to peer recognition. They often share drinks, snacks or fried food in their interaction with peers, which is contrary to the implementation of healthy and nutritional behavior, but it is a strategy for students to establish good interpersonal relationships. As the scholar Wilkerson (47) pointed out, eating behavior will be affected by interpersonal and reference groups, especially peer eating behavior (snacks or sweets). In fact, diet for people is not only the intake of nutrition, but also has more deep significance, such as social and cultural. Therefore, in the intervention of nutritional behavior, we should not only consider nutritional behavior, but should think more, find out the key factors and intervene effectively.

The third group of canonical correlation found that adolescent sports behavior is mainly affected by the sense of physical ability and physiological value. The higher the sense of physical ability and physiological value, the more able to implement sports behavior. In this study, the sense of physical ability and physiological value means that individuals evaluate and feel themselves in body image, appearance and physical fitness. The higher the sense of physical ability and physiological value, it means that they have a positive evaluation and feeling on their body image, appearance and physical fitness. In other words, the more positive the evaluation and feeling of their body image, appearance and physical fitness, the more willing they are to engage in sports behavior. This reaction echoes the previous studies (48–50) on the impact of self-esteem on exercise behavior. It can be seen that in order to improve the implementation of students' sports behavior, in addition to the improvement of sports knowledge, ability and technology, it is also very important to improve their self-esteem and body image.

The fourth group of canonical correlation found that external recognition and physiological value affect adolescents' nutritional behavior and stress management. The lower the external recognition and the higher the sense of physiological value, the higher the performance of nutritional behavior and the better the stress management. In this study, external recognition refers to the degree to which individuals rely on external recognition and approval to evaluate their self-ability and value. The results show that the more adolescents do not rely on external recognition to evaluate their self-ability and value, and have positive evaluation and feelings on their body image, appearance and physical fitness, the more they can implement nutritional behavior and better stress management, These findings also echo the previous research conclusions of relevant scholars (51, 52). In addition, the typical correlation of this group also found that the physiological value not only affects the implementation of sports behavior, but also affects the implementation of nutritional behavior. The higher the physiological value, the higher the performance of nutritional behavior and sports behavior. This finding can be used in the weight control intervention plan for adolescents, because a good weight control plan can achieve twice the result with half the effort if it can improve its physiological value and physical self-esteem at the same time, in addition to combining a balanced diet and regular exercise. Schwager et al. (3) conducted an intervention study on weight control of adolescent overweight girls and found that overweight girls have low self-esteem and 80% have depression tendency. Through diet cognition and exercise courses, they not only reduce weight, but also improve self-esteem. In this regard, relevant longitudinal empirical research needs to be done in the future to further confirm the relationship between self-esteem and diet and exercise behavior.

The fifth group of canonical correlation found that internal evaluation will affect adolescents' health responsibility, exercise behavior and stress management. The higher the internal evaluation, the more they can implement responsible behavior and sports behavior, and the better their stress management. In this study, the connotation of internal evaluation refers to an internally oriented and flexible evaluation orientation. Individuals with high self-esteem can rely on internal needs and standards to act and evaluate themselves. In other words, the more people can act and evaluate themselves according to their internal needs and standards, the more they can implement responsible behavior and sports behavior, and the better their stress management. This research result is consistent with Robinson's view that (53, 54) whether human behavior repeats or not is affected by two factors: one is the value of the reinforcement in the individual's mind, the other is the individual's possibility evaluation and expectation of obtaining the reinforcement, and believes that the individual's control over the reinforcement is internal and external, that is, the internal controller and the external controller, and the external control believer believes that, The acquisition of reinforcement has nothing to do with one's own behavior, and will be controlled by luck, opportunity, destiny, powerful others or unknown forces in the external environment; The internal controller will think that the acquisition of reinforcement is caused by his own behavior or some quality, ability and characteristic. It can be seen that internal control students can better implement responsible behavior, sports behavior and stress management. Therefore, the findings of this group remind us that in order to more effectively implement the promotion of teenagers' health responsibility, sports behavior and stress management, we should pay attention to the internal needs of students rather than external norms.

## Conclusion

1) Girls are more able to implement health responsibility and nutritional behavior than boys, while boys are more able to implement stress management, social support and sports participation than girls; Boys have higher control perception than girls, while girls have higher evaluation orientation than boys; From the overall perspective of self-esteem, boys's perception of self-esteem is not as good as girls, while from the overall behavior of health promotion, boys are more likely to implement health promotion than girls.

2) Compared with children from single parent or combined families, children from two parent families can not only implement health promotion behavior, but also have a higher degree of self-esteem; The higher the education level of parents, the better their children have health responsibility, higher sense of ability, value, evaluation orientation and overall self-esteem; Family economic status has little effect on adolescent health promotion and overall self-esteem.

3) The higher the self-esteem of adolescents, the more they can implement the health promotion behavior. Adolescents with a higher sense of interpersonal ability, physical ability, family ability, academic ability, family control, control, academic control, internal evaluation, interpersonal value, physiological value, family value and academic value will implement the health promotion behavior better, and the higher the sense of powerlessness, The worse the implementation of health promotion behavior.

4) The higher the adolescents' sense of interpersonal ability, the more they can implement social support behavior; The higher the sense of physical ability and physiological value, the more able to implement sports behavior; The higher the sense of academic ability, the more able to implement nutritional behavior; The higher the external recognition and physiological value, the better the nutritional behavior and stress management; The higher the internal evaluation, the more able to implement health responsibility, exercise behavior and stress management behavior.

## Data Availability Statement

The original contributions presented in the study are included in the article/supplementary material, further inquiries can be directed to the corresponding authors.

## Ethics Statement

This study was reviewed and approved by the ethics review committee of Southwest University. However, this study does not involve human and animal experiments, and written informed consent is not required.

## Author Contributions

BL is mainly responsible for research design and providing financial support for this research. At the same time, he also participated in some writing of the paper. SY and XW are mainly engaged in the distribution of the questionnaire and data processing and analysis. JL is responsible for the overall correction, revision, translation and posting of the thesis. All authors contributed to the article and approved the submitted version.

## Funding

This study is supported by the National Social Science Foundation of China. Project No.: 19BTY073.

## Conflict of Interest

The authors declare that the research was conducted in the absence of any commercial or financial relationships that could be construed as a potential conflict of interest.

## Publisher's Note

All claims expressed in this article are solely those of the authors and do not necessarily represent those of their affiliated organizations, or those of the publisher, the editors and the reviewers. Any product that may be evaluated in this article, or claim that may be made by its manufacturer, is not guaranteed or endorsed by the publisher.
